# Historic evolution of population exposure to heatwaves in Xinjiang Uygur Autonomous Region, China

**DOI:** 10.1038/s41598-023-34123-w

**Published:** 2023-05-06

**Authors:** Diwen Dong, Hui Tao, Zengxin Zhang

**Affiliations:** 1 College of Ecology and Environment, Xinjiang University, Urumqi, 830046 China; 2 State Key Laboratory of Desert and Oasis Ecology, Xinjiang Institute of Ecology and Geography, Chinese Academy of Sciences, Urumqi, 830011 China; 3 University of Chinese Academy of Sciences, Beijing, 100049 China; 4 College of Statistics & Data Science, Xinjiang University of Finance & Economics, Urumqi, 830012 China; 5 Joint Innovation Center for Modern Forestry Studies, College of Forestry, Nanjing Forestry University, Nanjing, 210037 Jiangsu China

**Keywords:** Climate sciences, Natural hazards

## Abstract

Heatwaves have pronounced impacts on human health and the environment on a global scale. Although the characteristics of heatwaves has been well documented, there still remains a lack of dynamic studies of population exposure to heatwaves (PEH), particularly in the arid regions. In this study, we analyzed the spatio-temporal evolution characteristics of heatwaves and PEH in Xinjiang using the daily maximum temperature (T_max_), relative humidity (RH), and high-resolution gridded population datasets. The results revealed that the heatwaves in Xinjiang occur more continually and intensely from 1961 to 2020. Furthermore, there is substantial spatial heterogeneity of heatwaves with eastern part of the Tarim Basin, Turpan, and Hami been the most prone areas. The PEH in Xinjiang showed an increasing trend with high areas mainly in Kashgar, Aksu, Turpan, and Hotan. The increase in PEH is mainly contributed from population growth, climate change and their interaction. From 2001 to 2020, the climate effect contribution decreased by 8.5%, the contribution rate of population and interaction effects increased by 3.3% and 5.2%, respectively. This work provides a scientific basis for the development of policies to improve the resilience against hazards in arid regions.

## Introduction

Heatwaves are generally defined as extended periods of extremely high temperatures. It can have catastrophic impacts on human health, infrastructure, agricultural ecosystems, and the national economy^1–3^. For example, the intense heatwaves resulted in more than 70,000 deaths in Europe in 2003^4^, the railway infrastructure in Australia was damaged in 2009^5^, and the crop failure in Russia in 2010 was about 25%^6^. Especially, in recent years, China has been hit by heatwaves several times. Shanghai and Xinjiang suffered unprecedented heatwaves in 2013 and 2015, respectively^7,8^. According to the 2020 China report of The Lancet Countdown on health and climate change, heatwave-related mortality has a four-fold increase from 1990 to 2019 in China^9^. Under the background of global climate change, the adverse impacts of increasing heatwaves on human health and socio-economics will become more serious^10,11^. However, few studies explored the spatial heterogeneity of heatwaves and the dynamic variation of population exposure simultaneously. Thus, there is an urgent requirement to elucidate the spatio-temporal evolution of heatwaves and quantify the deleterious impacts of heatwave exposure for providing scientific insights for coping with climate change.

Currently, heatwaves have attracted widespread attention from government departments and the scientific community. Most heatwave investigations includes limited metrics such as frequency, intensity, and duration^12,13^. Prolonged exposure to heatwaves can endanger human health^14^, thus more detailed studies of the adverse effects of heatwaves on population is required^15^. Previous studies have typically linked heatwaves to human health through heat-related morbidity and mortality data^16,17^. Hajat et al.^18^ investigated that death rate increased by 3.34% for every degree of temperature increase. Nevertheless, the heat-related mortality is related to climate and population. Jones et al.^19^ calculated population exposure to heatwaves spatially, in other to quantify the influence of natural hazards. It is difficult to deeply analyze the dynamics of spatial population exposure on the basis of previous population data by administrative regions. This limitation can be effectively overcome by high-resolution gridded population datasets^20^.

Heatwaves in the Xinjiang Uygur Autonomous Region have increased significantly over the last few decades^21,22^, being expected to occur more continually and intensely in the future^23^. Previous studies in Xinjiang identifies heatwaves by daily maximum. In addition to the high temperature, human comfort and health are also affected by the relative humidity (RH), wind speed and the solar radiation^24,25^. Furthermore, as the RH increases, some heat-related deaths happen at relatively low temperatures, illustrating that the RH play another significant role in identifying heatwaves^26,27^. Currently, the climate in Xinjiang has exhibited a trend of ‘warming-wetting’^28,29^. There might be great uncertainty when attempting to estimate heatwaves without considering the RH. Hence, the indices combining temperature and RH are more suitable for characterizing heatwaves in Xinjiang.

Compared with the characteristics of heatwaves, few studies have focused on the population exposure to heatwaves (PEH) in Xinjiang. Even though some relevant studies in large scale regions have included Xinjiang, the assessment results do not accurately reflect the evolution of PEH in Xinjiang^30^. Xinjiang is at the heart of the Belt and Road Initiative, with higher population growth than the national average. Therefore, studies on the PEH in Xinjiang are necessary. The spatial distribution of population in Xinjiang is influenced by the oasis and typical of the population distribution in arid areas^31^. Studying the characteristic of heatwave and PEH in Xinjiang can provide a scientific basic for active response to climate change in arid regions.

In this paper, we investigate the dynamic changes of heatwaves and PEH using a long time series of high-resolution population data and further quantify the factors driving the PEH. The primary goal of this study includes: (1) revealing the spatial and temporal evolution of heatwaves under the ‘warming-wetting’ trends in Xinjiang, (2) assessing the dynamic changes of population exposure under the rapidly increasing heatwaves and population in Xinjiang, and (3) quantifying the impacts of climate and population changes on the PEH in Xinjiang. This work provides the useful scientific support for the design of strategies to deal with climate change in Xinjiang.

## Results

### Interannual variability of heatwave

**Figure 1:**
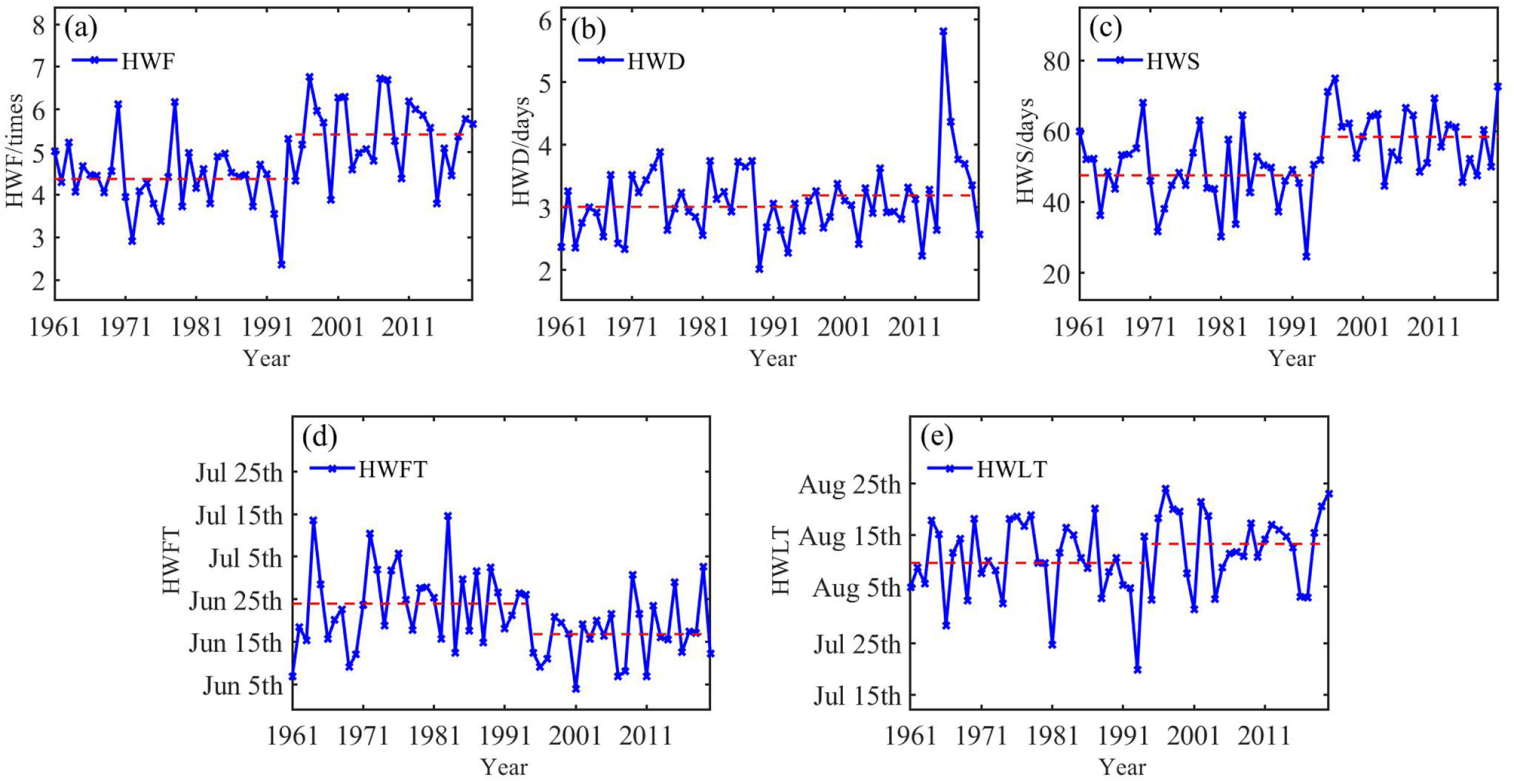
Times series of (**a**) heatwave frequency (HWF), (**b**) heatwave duration (HWD), (**c**) heatwave season length (HWS), (**d**) first heatwave timing (HWFT), and (**e**) last heatwave timing (HWLT) in Xinjiang from 1961 to 2020. Dashed lines indicate their average of the corresponding time period.

The temporal variability of annual heatwave in Xinjiang for the period 1961–2020 is presented in Fig. 1. The heatwave frequency (HWF) (Fig. 1a) and heatwave season length (HWS) (Fig. 1c) shows fluctuating changes with an abrupt change around 1994. The mean values for HWF and HWS increases by 1.04 times and 10.9 days after mutation, respectively. For the heatwave duration (HWD) (Fig. 1b), no significant change is noticed for the whole series and 2015 is the year with the longest HWD (5.8 days). Besides the increase HWF and HWD, the increase in heatwaves is also associated with the changes in first heatwave timing (HWFT) (Fig. 1d) and last heatwave timing (HWLT) (Fig. 1e). The HWFT begins mainly in June and the difference between the earliest and the latest onset year is 41 days. While the HWLT ends in August for most years. The latest year for the end of HWLT between 1961 and 2020 is 1997. In general, not only the HWF and HWS are increasing in Xinjiang, but the HWFT is advancing and HWLT is postponing.

### Heatwave spatial distribution and variations

**Figure 2:**
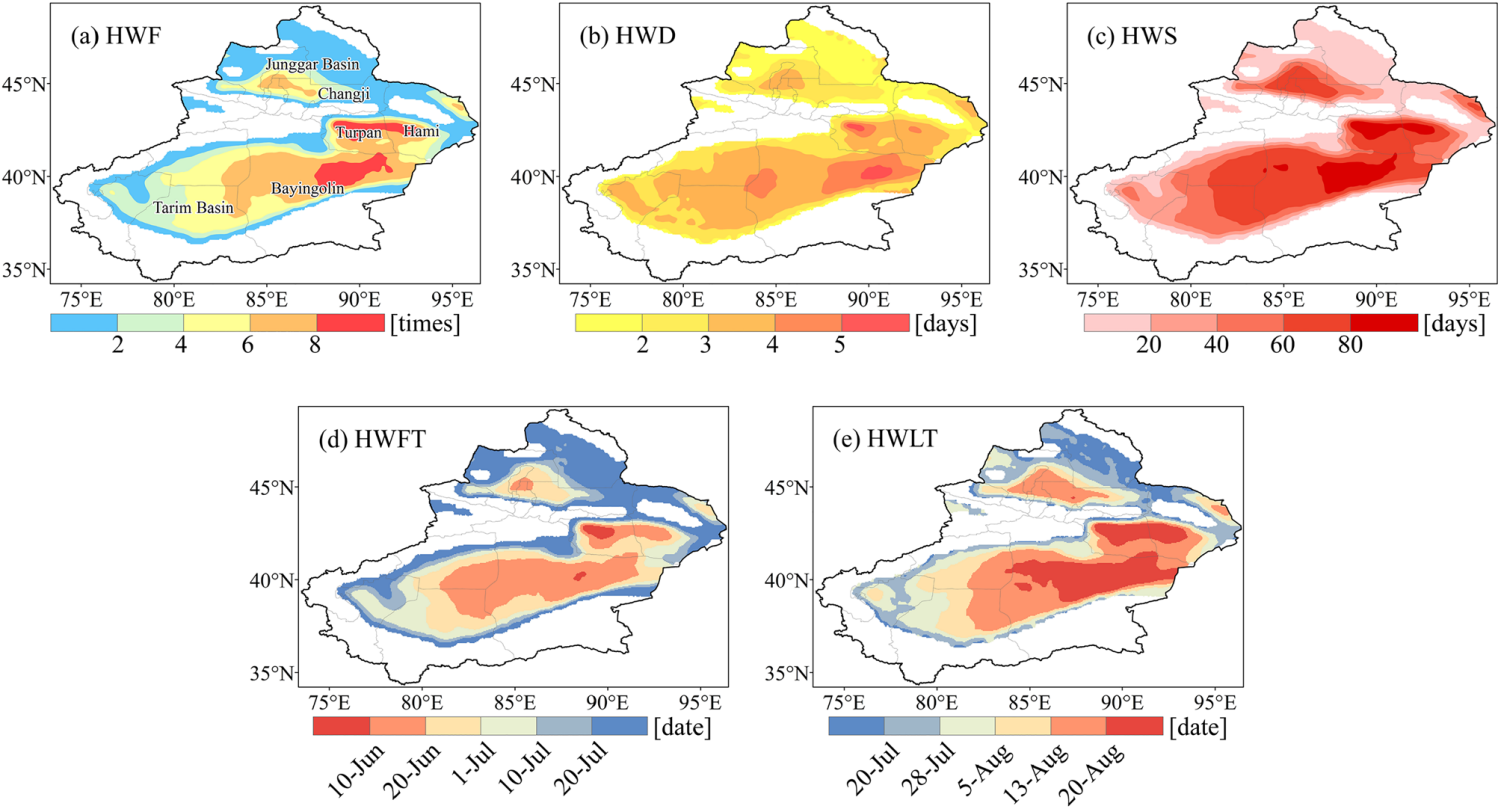
Spatial distribution of annual averaged (**a**) heatwave frequency (HWF), (**b**) heatwave duration (HWD), (**c**) heatwave season length (HWS), (**d**) first heatwave timing (HWFT), and (**e**) last heatwave timing (HWLT) in Xinjiang from 1961 to 2020.

Heatwaves have occurred in most areas of Xinjiang from 1961 to 2020. The distribution of heatwaves exhibits significant spatial heterogeneity. The high values of HWF (Fig. 2a), HWD (Fig. 2b), and HWS (Fig. 2c) are concentrated in Turpan, Hami, and the eastern portion of Bayingolin. Simultaneously, the HWFT begins earliest and the HWLT ends latest in these areas (Fig. 2d,e). The low values are broadly distributed in Altay, the margin of the Junggar Basin, and the margin of the Tarim Bain. High-altitude mountains such as the Kunlun, Tianshan, and Altai Mountains have never been hit by heatwaves from 1961 to 2020. Our study revealed that most heatwaves in Xinjiang are occurred in areas below the altitude of 1500 m, and never occurred in areas above the altitude above 2500 m.

**Figure 3:**
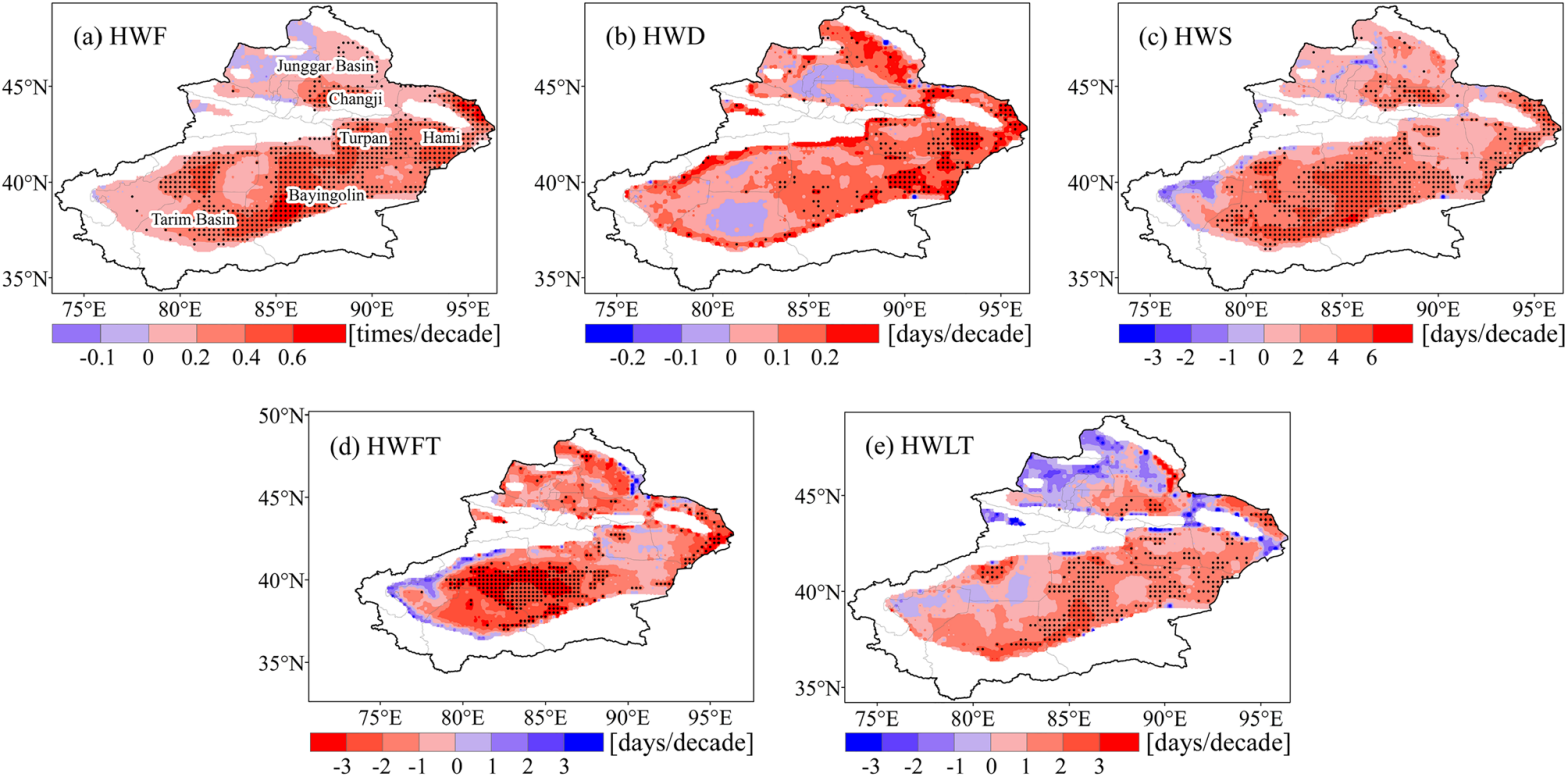
Trends of (**a**) heatwave frequency (HWF), (**b**) heatwave duration (HWD), (**c**) heatwave season length (HWS), (**d**) first heatwave timing (HWFT), and (**e**) last heatwave timing (HWLT) in Xinjiang from 1961 to 2020. Stippling denotes statistically significant trends (*p* < 0.05).

Figure 3 shows the spatial pattern trends in heatwaves. Significantly increasing trends (*p* < 0.05) in both the HWF (Fig. 3a) and HWS (Fig. 3c) can be observed in many areas of Xinjiang, such as the Tarim Basin, Changji, Hami and the Southern Junggar Basin. However, the remarkable increase of the HWD (Fig. 3b) are concentrated in Hami and the eastern portion of Bayingolin. Similarly, the advanced HWFT (Fig. 3d) and the delayed HWLT (Fig. 3e) are most notable in the Tarim Basin. Overall, heatwaves are more frequent, longer lasting, and more intense in most areas of Xinjiang.

### Heatwave grades

**Figure 4:**
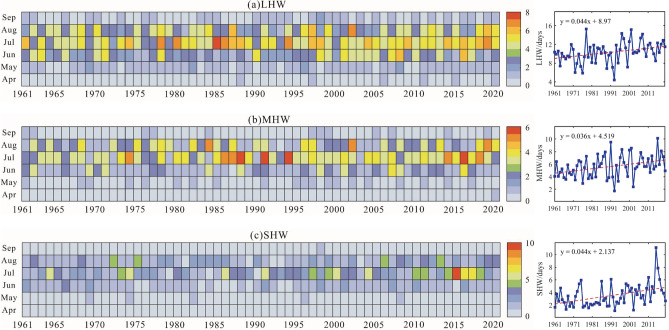
Times series of (**a**) light heatwaves (LHW), (**b**) moderate heatwaves (MHW), and (**c**) strong heatwaves (SHW) in Xinjiang from 1961 to 2020. Dashed lines indicate corresponding linear trends.

From 1961 to 2020, heatwaves in Xinjiang typically occurred between May and August (Fig. 4). In terms of occurrence times, the light heatwaves (LHW) (Fig. 4a) occurrs earliest (beginning on May 21 on average), which is 13 and 29 days earlier than the appearance of moderate heatwaves (MHW) (Fig. 4b) and strong heatwaves (SHW) (Fig. 4c), respectively. Meanwhile, the LHW is the latest ending (August 31 on average). From the perspective of the area and duration, Xinjiang is primarily affected by LHW, followed by MHW, and the least by SHW. However, 2015 is a very unique year. The number of SHW days not only the highest from 1961 to 2020 (8.2 days), but more than the number of MHW days in 2015.

**Figure 5:**
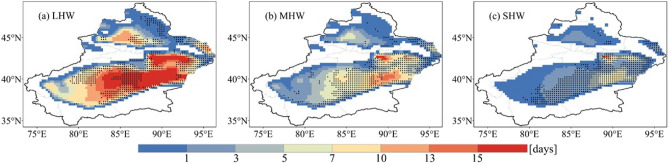
Spatial distribution of (**a**) light heatwaves (LHW), (**b**) moderate heatwaves (MHW), and (**c**) strong heatwaves (SHW) in Xinjiang from 1961 to 2020. Stippling denotes statistically significant trends (*p* < 0.05).

Different grades of heatwave days are increasing significantly in most parts of Xinjiang, regardless of the grade (Fig. 5). The areas of significant increase in LHW are widely distributed in Aksu, Bayingolin, Changji, Turpan, and Hami. While the areas where MHW and SHW increase significantly are concentrated in Bayingolin, Turpan, and Hami. The area of heatwaves affected by different grades is LHW > MHW > SHW. In other words, areas where LHW occurs do not necessarily have MHW and SHW, while areas where SHW occurs must have had LHW. However, this indicates that not all areas where heatwaves occurred are hit by SHW, which is particularly obvious in Yili and Altay.

### Analysis of PEH and its influencing factors

**Figure 6:**
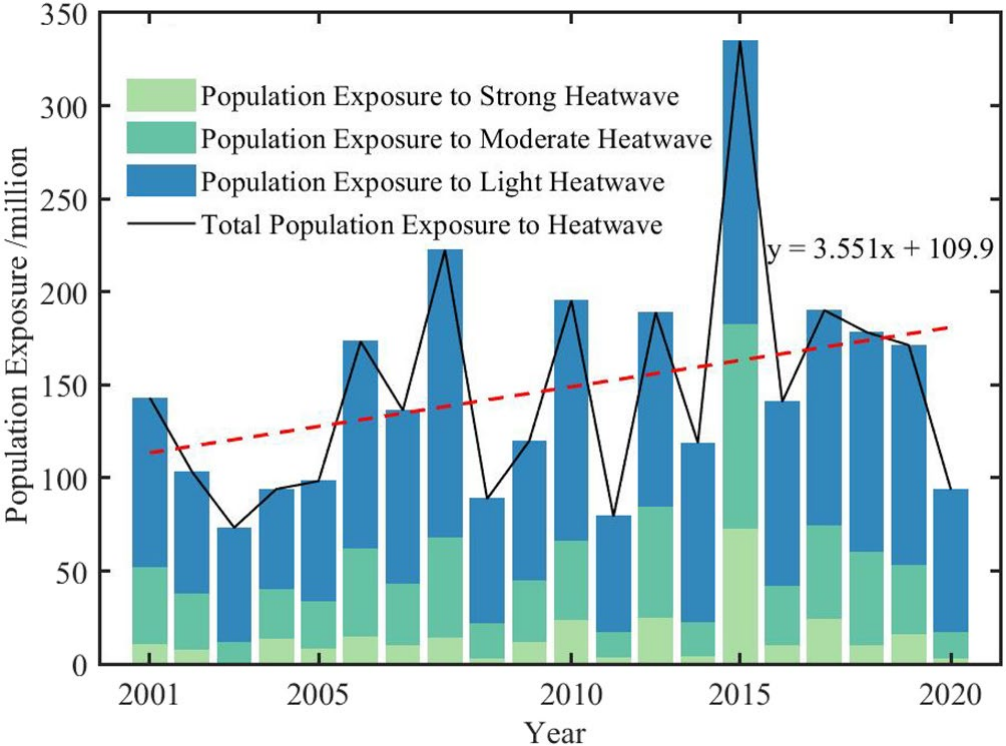
Population exposure to different grades of heatwaves in Xinjiang from 2001 to 2020. Dashed line indicates corresponding linear trends.

Exposure is an important indicator for disaster risk assessment, which is of great significance for conducting high-temperature warning work^32^. It can be observed from Fig. 6 that the PEH in Xinjiang is primarily affected by the LHW, and less affected by the MHW and SHW. The total PEH exhibits an increasing trend from 2001 to 2020. The year with the highest PEH in Xinjiang is not 2020 with the largest population, but rather 2015 with the longest HWD. This indicates that in addition to population growth, climate change also have an impact on changes in the PEH.

**Figure 7:**
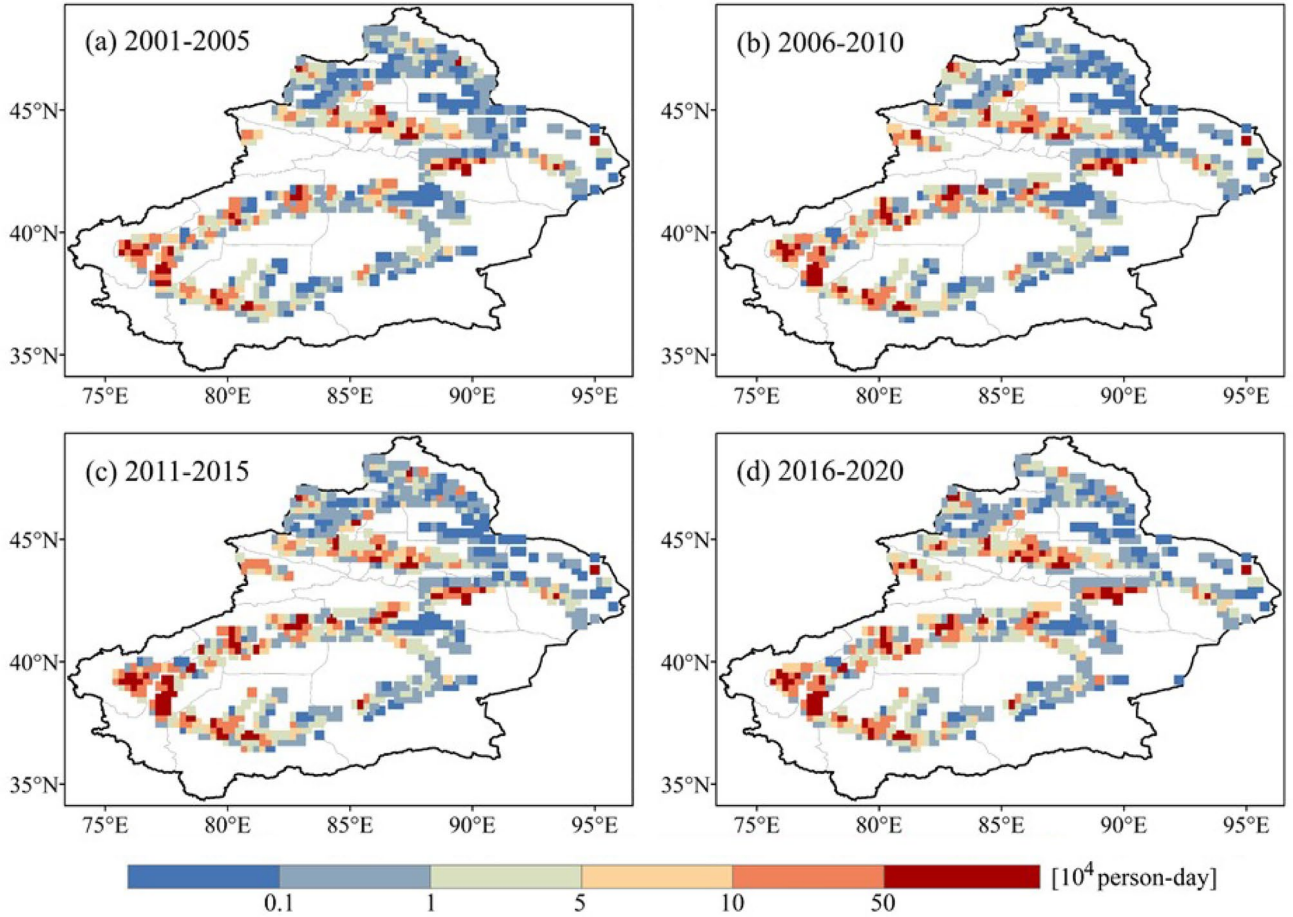
Spatial distribution of population exposure to heatwaves (PEH) in Xinjiang.

The spatial distribution of PEH shows significant geographical heterogeneity, which is consistent with the spatial distribution of the population (Fig. 7). The spatial pattern of PEH in Xinjiang is similar for different periods. Areas with high PEH (10^5^ person-days or more) are mainly located in Kashgar, Aksu, Turpan, and Hotan. The areas of extremely low PEH (10^3^ person-days and below) are broadly distributed in Altay, the margin of the Junggar Basin, and the eastern portion of the Tarim Basin. Although the hinterlands of the Tarim and Junggar Basins are subject to frequent heatwaves, there is no PEH due to the absence of a population.

**Figure 8:**
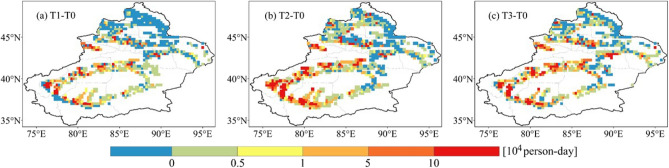
Changes in population exposure to heatwaves (PEH) for different periods (**a** 2006–2010, **b** 2011–2015, and **c** 2015–2020) compared with the base period (2001–2005).

To further investigate the variation of PEH in Xinjiang, we compared the PEH in other periods with the T0 period (Fig. 8). We found that in densely populated areas (e.g., Hotan, Kashgar, and Aksu), the increase in PEH is considerable. The PEH in Bayingolin do not increase noticeably, while it does decrease in Altay.

The increase in PEH is mainly contributed from population growth, climate change and their interaction. To explore the relative importance, we evaluated the rate of contribution to PEH changes for each factor. Table 1 indicates that the contribution rate of the climate effect tends to decrease over time (8.5%). Simultaneously, the contribution rate of the population and interaction effects gradually increased by 3.3% and 5.2%, respectively. In conclusion, the climate effect is dominant over population and interaction effects in Xinjiang.

**Table 1:** Contribution rates to population exposure changes for each factor.

Period	Population exposure change (10^6^) person-days	Contribution rate of influencing factors (%)		
		Climate effect	Population effect	Interaction effect
T1–T0	38	53.9	37.8	8.3
T2–T0	94	48.5	39.1	12.4
T3–T0	65	45.4	41.1	13.5

### Heatwave in typical year

**Figure 9:**
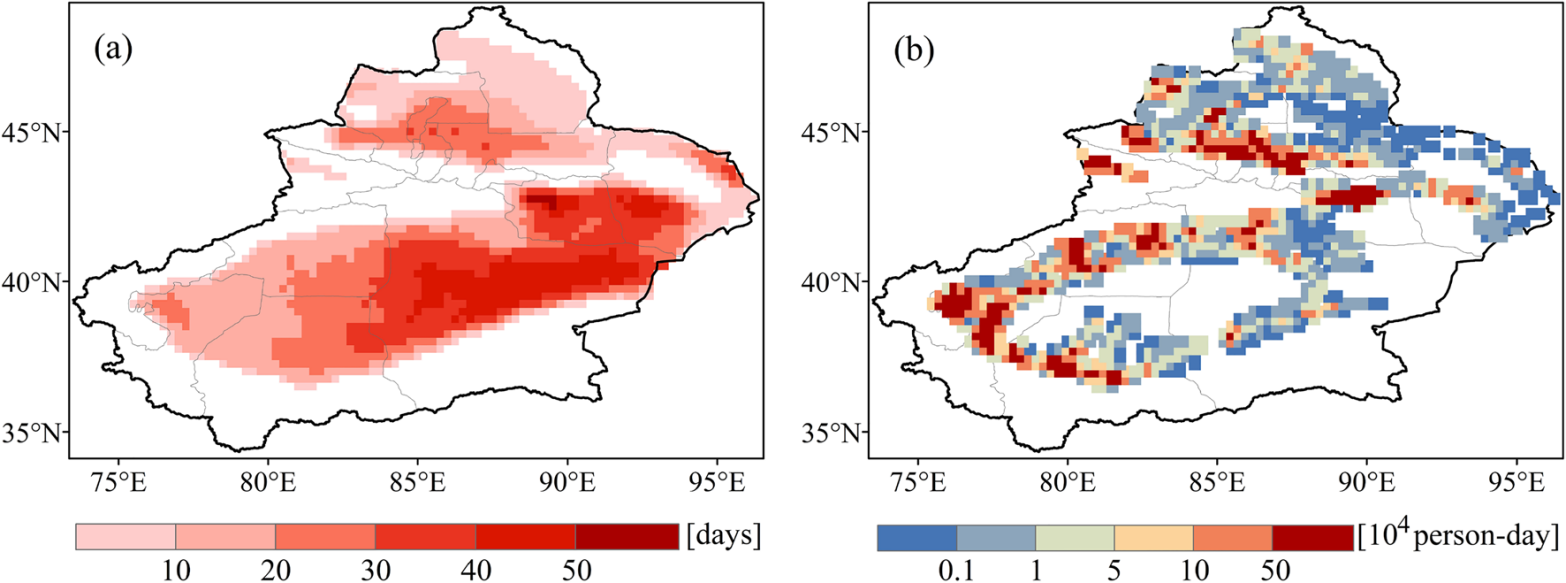
(**a**) Heatwave days in Xinjiang for 2015. (**b**) Population exposure to heatwaves in Xinjiang for 2015.

**Figure 10:**
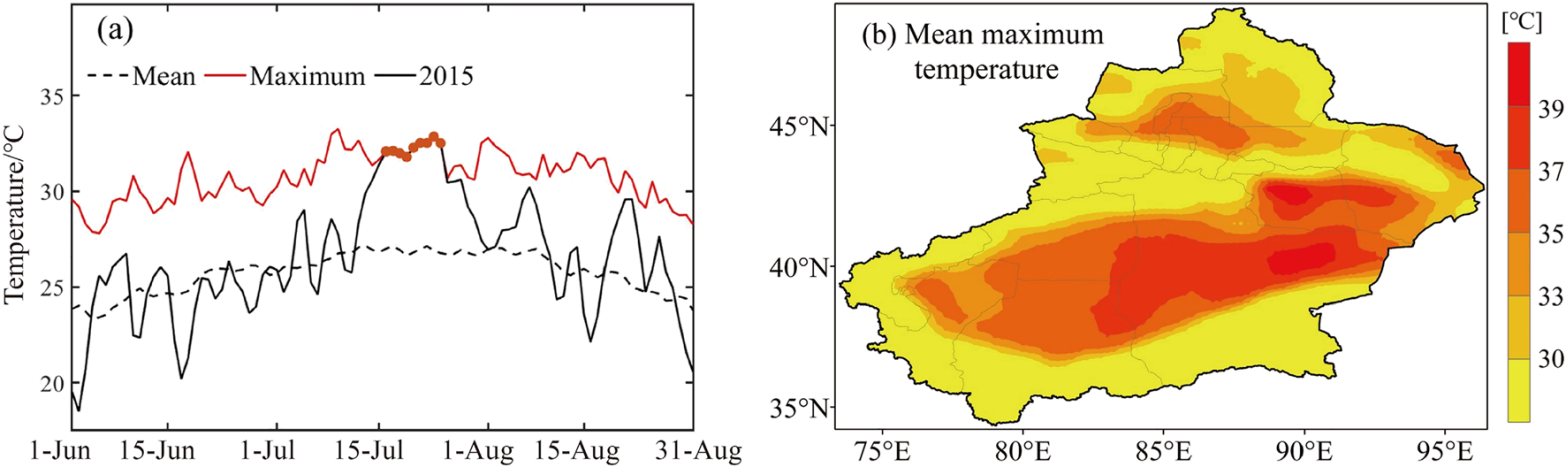
(**a**) Variation of daily maximum temperature (T_max_) in Xinjiang. The red solid line represents the maximum values of T_max_ from 1961 to 2020. The black solid line represents the maximum values of T_max_ in 2015. Red dots represent the maximum values of Tmax in 2015 that is the maximum from 1961 to 2020. Black dashed line represents T_max_ averaged from 1961 to 2020. (**b**) Spatial distribution of the T_max_ in Xinjiang for July 2015.

Xinjiang suffered the severest heatwave and the highest PEH in 2015 (Fig. 9). Figure 10 shows clearly that high temperatures occurred widely in Xinjiang in 2015 and also concentrated in mid to late July. Consecutive days of Tmax exceeded the historical day, which is consistent with the results of this study.

**Figure 11:**
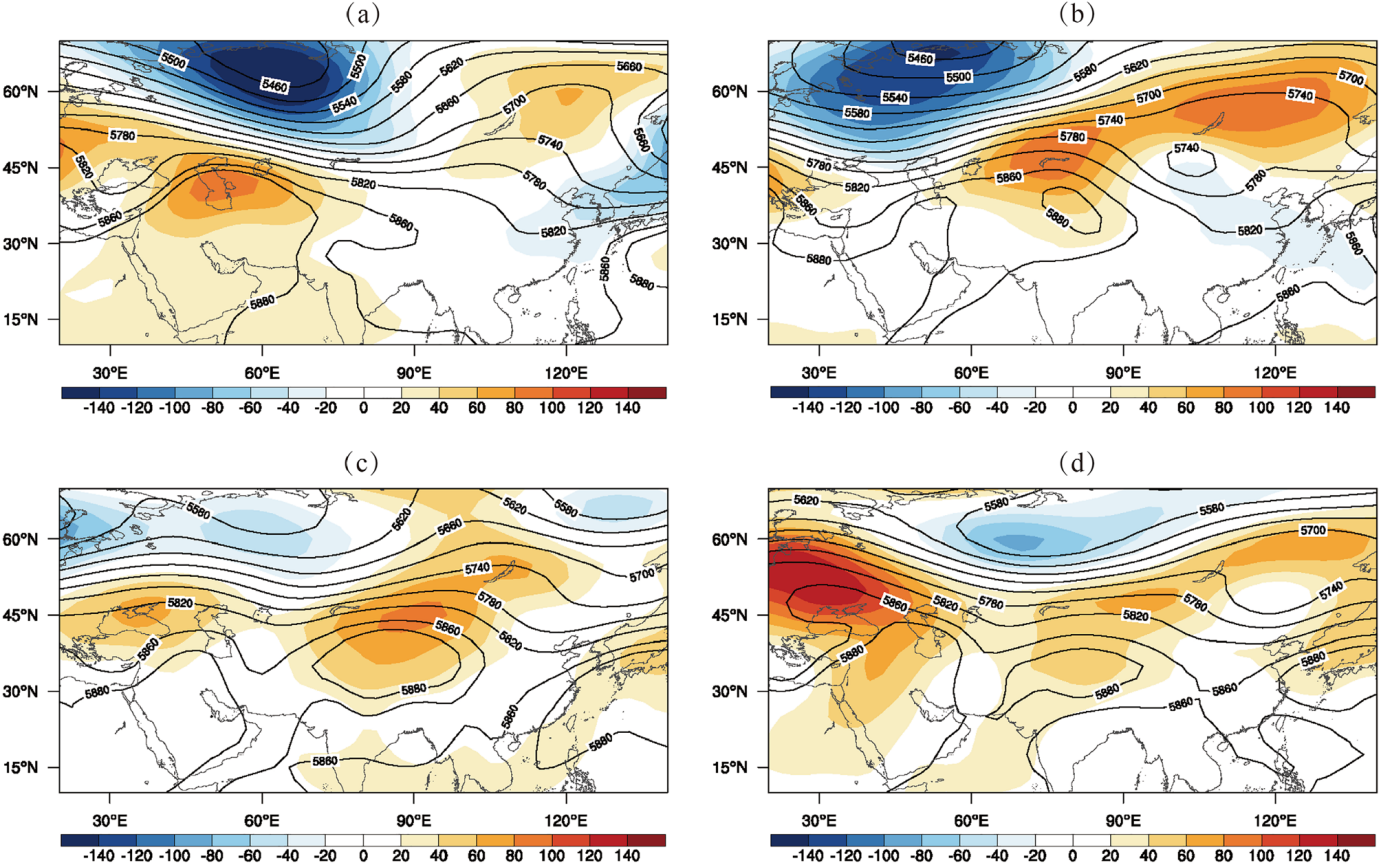
The geopotential height fields in (**a**) early July, (**b**) mid-July, (**c**) late July, and (**e**) early August for 2015.

Xinjiang is located at the mid-latitudes in the Northern Hemisphere, where temperatures will continue to rise under the steady control of high-pressure systems^8^. Figure 11 illustrates the evolution of the geopotential height field at 500 hPa from July to early August, 2015. We find the center of high pressure over the Iranian plateau shifts eastwards to Xinjiang, then remains stable and gets strengthened over Xinjiang, followed by the southward shift and getting weakened. The evolution of this Iranian high pressure coincides with corresponding changes in the high temperatures in Xinjiang. Therefore, the eastward shift of Iranian high pressure and its control of Xinjiang is the direct cause of this heatwave.

## Discussion

This study examines the dynamic changes of heatwaves and PEH using a long time series of high-resolution population data and further quantifies the factors causing the PEH changes. By analyzing heatwave metrics, the spatio-temporal evolution pattern of heatwaves in Xinjiang has been comprehensively captured. The abrupt change point of heatwaves in 1994 is observed, coinciding with that in annual Tmax of Xinjiang occurring in the 1990s^33^. Chen et al.^34^ discovered an increase in the number of high-temperature days in Xinjiang when the South Asian high (SAH) is strong and its central area is located northwards. The increase in high temperatures could trigger the corresponding increase in relative humidity. This could have an impact on heatwaves in these areas. China suffered severe heatwaves due to a super El Niño event that occurred in 1997^35^, as well as the maximum values of HWF and HWS in Xinjiang occurred in the same year. This suggests that changes in heatwaves in Xinjiang is linked to El Niño event.

Because of the uneven global warming, the distribution of heatwaves have significant spatial heterogeneity. In this study, we found that the Eastern Tarim Basin, Turpan, and Hami are the most heatwave-prone, with the most significant increases in heatwaves. This is not only related to the high temperatures due to the low altitudes, also correlated with atmospheric circulation^36^. For example, Mao et al.^8^ discovered the eastward shift of Iranian high pressure led to the longest lasting and most widespread heatwave of Xinjiang in 2015. In addition, the distribution of heatwaves is influenced by land use and latitude^37,38^. Heatwaves in northern Xinjiang are mainly distributed in the Junggar Basin. This aligns with the conclusion of Xin et al.^39^ that altitude is the main factor influencing the distribution of high temperature, followed by latitude.

The timing of heatwaves is another important feature. Besides the HWF, HWD, and HWS, we also examined the HWFT and HWLT in Xinjiang from 1961 to 2020. Early heatwaves may have had a greater negative impact on human health than later heatwaves. This may be due to the fact that human have little opportunity to acclimatize to them^40^. It is revealed that the HWFT in Xinjiang occurs mostly from early June to early July. Compared with other regions in China, the heatwave occurrence dates in Xinjiang are the same as those in North China, later than those in Southwestern China, and earlier than those in South China^41^. Generally, although the start of HWFT and the end of HWLT vary in different areas of Xinjiang, the HWLT ends later in those areas where the HWFT appears earlier. With the advanced HWFT and delayed HWLT, the impacts of heatwaves on human health are exacerbated and economic losses will increase^42^. For example, Mao et al.^8^ found that high temperatures caused ice melt flooding in the Tarim River Basin in 2015, and resulted in various degrees of damage to rail and road traffic, as well as water facilities.

Heatwaves are graded according to the magnitude of the HI, and it is revealed that the number of heatwave days increases from 1961 to 2020, regardless of the heatwave grade. In terms of the number of days and affected areas, the LHW is the most, followed by MHW, and SHW is the least. However, the number of SHW days in 2015 is not only the most for the period of 1961–2020, but also more than the number of MHW days in that year. This impacts the yields and quality of growing crops such as corn, cotton, and grapes^43^. Also, the number of patients with airway diseases and cerebrovascular diseases due to heatwaves increased significantly. According to the study of Liu et al.^21^, with the intensification of global warming, heatwave affected areas will expand, and more severe heatwaves are likely to occur in the future. LHW may occur in regions that have not previously experienced heatwaves, whereas the original LHW may be transformed to MHW or even SHW. An increase in the different grades of heatwaves in the future may have serious deleterious impacts on agricultural livelihoods and human health in Xinjiang.

Heatwaves have been responsible for more annual deaths than other natural disasters in many areas of the world^44^, and the PEH in Xinjiang shows an increasing trend. Influenced by the geographic pattern of ‘mountain-oasis-desert’, the PEH is not distributed in the desert areas where heatwaves are frequent but concentrated in the oasis regions^45^. Areas with high PEH (10^5^ person-days or more) are primarily located in the Kashgar, Aksu, and Hotan, mainly because these areas are predominantly agricultural and have a dense population distribution^31^. Factors influencing changes in the PEH vary with regions^46^. In Europe, where population changes are relatively small or are even decreasing, the climate effect is the predominant driver for the increases in exposure. Whereas in many parts of Oceania and North Asia, changes in the PEH are mostly the result of population effects^47^. Our study shows that the PEH changes in Xinjiang are mostly dominated by the climate effect, followed by the population effect. This is consistent with the finding that the highest PEH is not in 2020 with the highest population, but in 2015 with the longest HWD.

## Methods

### Study area

**Figure 12:**
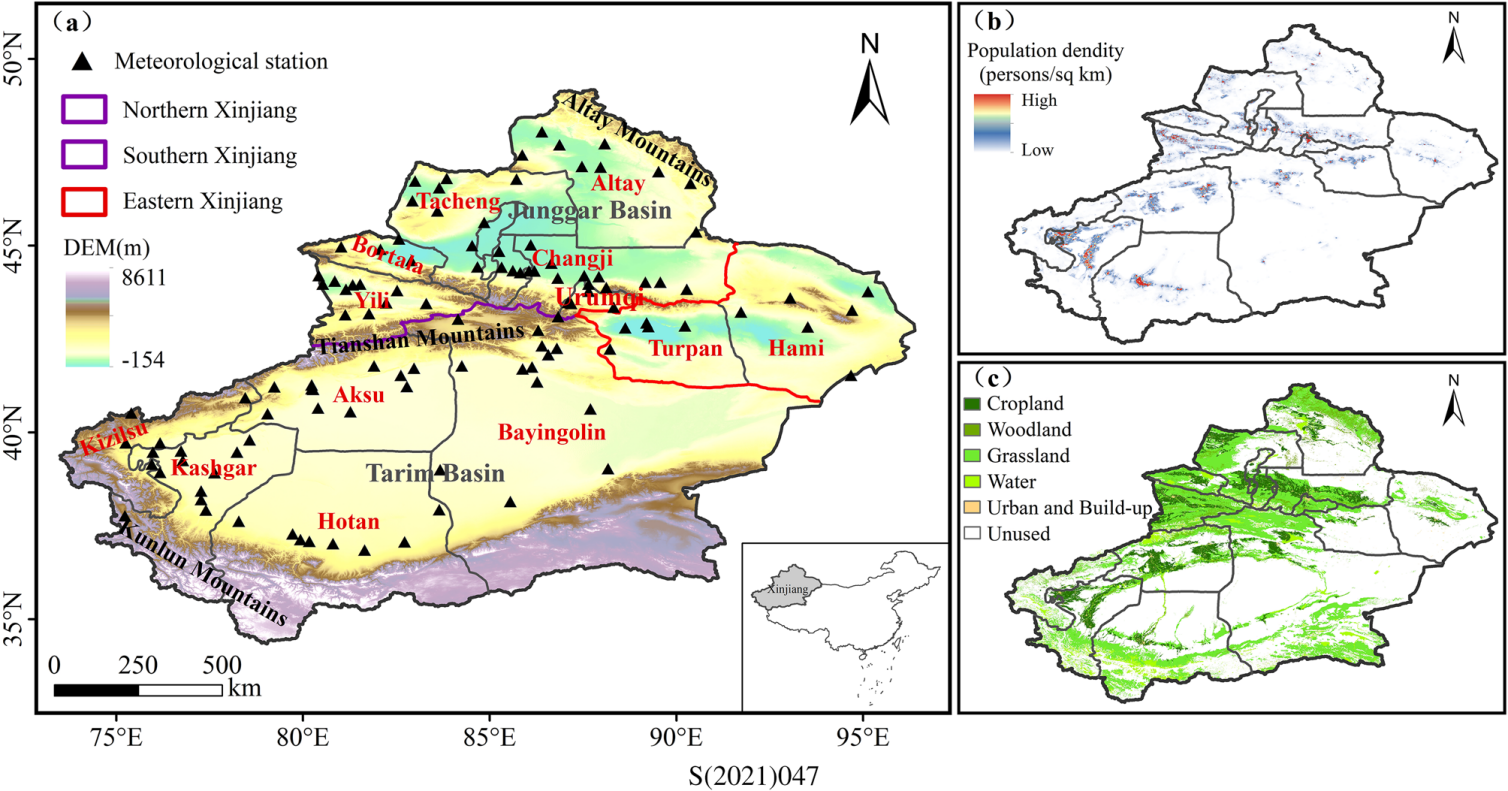
Maps of the study region. (**a**) Topographic features of Xinjiang and subregional divisions: Northern Xinjiang, Southern Xinjiang, and Eastern Xinjiang. (**b**) Population density. (**c**) Vegetation types.

The Xinjiang Uygur Autonomous Region (34° N–50° N, 73° E–96° E) is situated in Northwestern China (Fig. 12) with an area of approximately 1.6 million km^2^, which is the largest province in China. ‘Three Mountains and Two Basins’ is the major geographic feature of Xinjiang, with the Altai Mountains on the northern boundary and Kunlun Mountains on the southern boundary. The Tianshan Mountains between them is the natural geographical dividing line between the Junggar and Tarim Basins. It is customary to refer to the north of the Tianshan Mountains as Northern Xinjiang, the south as Southern Xinjiang, and Turpan and Hami as Eastern Xinjiang. Since Xinjiang is deep inland and far from the sea, it is highly sensitive to climate change^48^. The region has the typical arid and semi-arid continental climate, with scarce water resources, strong solar radiation, intense evaporation, and a fragile ecological environment^49^. It is extremely hot in the summer, with the highest temperature recorded as 50.5 ℃ in 2017.

Population growth is essential for economic and social development. The population of Xinjiang has grown steadily in recent years. According to national censuses data, the population of Xinjiang was 18.46 million in 2000 and 25.85 million in 2020, which is an increase of 7.39 million^50^. The distribution of the population in Xinjiang is influenced by the natural geographical pattern of ‘mountain-oasis-desert’, which is mainly concentrated in the oasis region^51^. Furthermore, due to the more developed economy and habitable climate in Northern Xinjiang, its population density is significantly higher than that of Southern Xinjiang and Eastern Xinjiang, where Urumqi, Turpan, Karamay, and Kashgar are densely populated.

### Data

The daily maximum temperature (Tmax) and relative humidity (RH) data from 1961 to 2020 are obtained from the China Meteorological Data Service Center (http://data.cma.cn). This dataset has a high spatial resolution of 0.25° × 0.25° and its quality control is strictly regulated by Wu et al.^52^. It is based on interpolations from over 2400 meteorological stations in China, where the climatology is initially interpolated by thin plate smoothing splines, after which a gridded daily anomaly is derive via the angular distance weighting (ADW) method and add to the climatology to obtain the final dataset. The standard map services are provided by the National Platform for Common Geospatial Information Services stand (https://www.tianditu.gov.cn).

**Figure 13:**
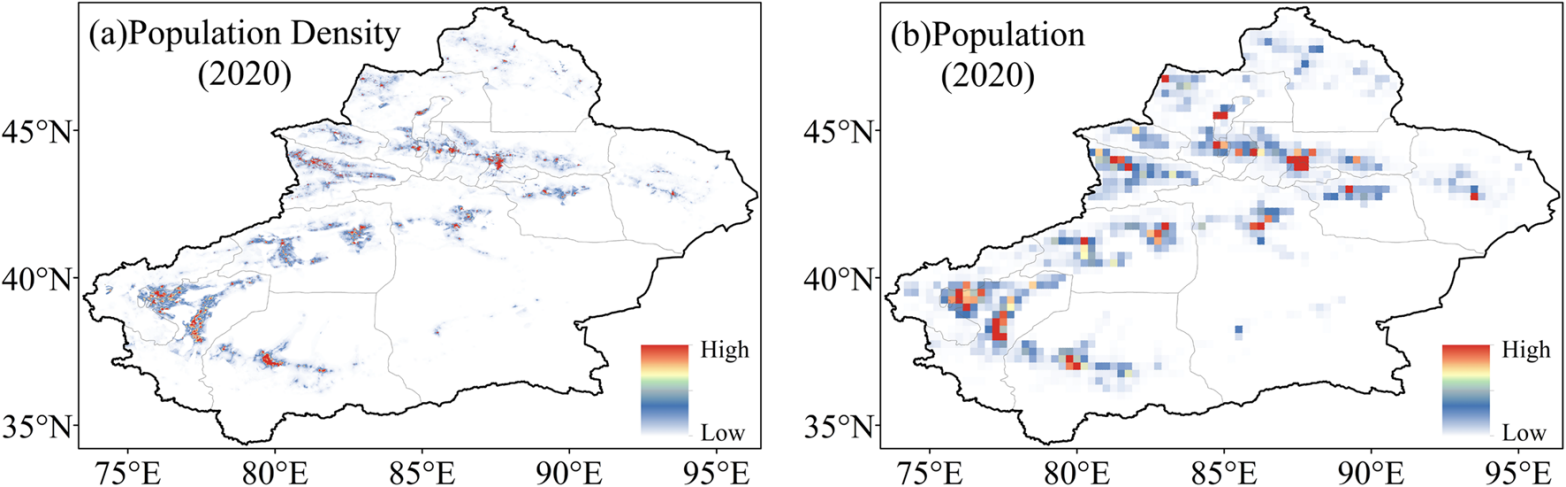
(**a**) Spatial patterns of population density in 2020. (**b**) Spatial patterns of population in 2020.

High-resolution gridded population dataset for 2001 to 2020 are obtained from the WorldPop (https://www.worldpop.org). The dataset translates official census data and a spatial auxiliary to a grid through a random forest model. The spatial auxiliary dataset includes settlement locations and ranges, satellite nighttime lighting data, land cover data, as well as road and building maps^53^. Many organizations and institutions employ the dataset for study since it is the most reliable long-term data series available^54,55^. Due to computational restraints, the Worldpop data are calculated as population in the 0.25° × 0.25° grid (Fig. 13).

### Methods

There is no established uniform definition of a heatwave due to the variable acclimatization and adaptability of certain population in different regions^47,56^. For this study, in consideration of the ‘warming-wetting’ climate trend in Xinjiang^57^, the heatwave index (HI) proposed by Huang et al.^58^ is used to identify heatwaves. The index is calculated by Eq. (1).

1 $${\text{HI}} = 1.2 \times ({\text{TI}} - {\text{TI}}^{\prime } ) + 0.35\sum\limits_{{i = 1}}^{{N - 1}} {1/{\text{nd}}_{{\text{i}}} ({\text{TI}}_{{\text{i}}} - {\text{TI}}^{\prime } )} + 0.15\sum\limits_{{i = 1}}^{{N - 1}} {1/{\text{nd}}_{{\text{i}}} } + 1$$

where $TI$ is the torridity index of the current day, ${TI}{^{\prime}}$ is the critical value of $TI$, ${TI}_{i}$ is the $TI$ of the next $\text{i}$ day before the current day, ${nd}_{i}$ is the number of days before the current day, and $N$ is the number of continuous hot weather days.

The torridity index for the RH (≤ 60%) is obtained using the following formula:

2 $${\text{TI}} = 1.8 \times {\text{T}}_{{\max }} - 0.55 \times \left( {1.8 \times {\text{T}}_{{\max }} - 26} \right) \times \left( {1 - 0.6} \right) + 32$$

For conditions where the RH is > 60%, the following formula is used:

3 $${\text{TI}} = 1.8 \times {\text{T}}_{{\max }} - 0.55 \times \left( {1.8 \times {\text{T}}_{{\max }} - 26} \right) \times \left( {1 - {\text{RH}}} \right) + 32$$

where ${T}_{max}$ is the daily maximum temperature (°C), $RH$ is the relative humidity (%).

The ${\text{TI}}{^{\prime}}$ is used to judge whether it is high temperature or hot weather. When ${\text{TI}}$ is greater than ${\text{TI}}{^{\prime}}$, it means that the given day reaches the high temperature state and is considered as hot weather. The quantile method is used to calculated ${\text{TI}}{^{\prime}}$, the following formulas are used:

4 $$\widehat{{Q}_{i}}\left(p\right)=\left(1-\gamma \right){X}_{\left(j\right)}+\gamma {X}_{\left(j+1\right)}$$

5 $$j = int\left[ {p \times n + \frac{{1 + p}}{3}} \right]$$

6 $$\gamma =p\times n+\frac{1+p}{3}-j$$

where $\widehat{{Q}_{i}}\left(p\right)$ is the $i$th quantile, $X$ denotes the sample sequence of the ${\text{TI}}$ in ascending order, $p$ is the 50% quantile, $n$ is the length of ${\text{TI}}$ series, $j$ is the $j$th ${\text{TI}}$, $\gamma$ is the weight of the $(j+1)$th number.

To measure the effects of various meteorological conditions on the socioeconomic status and human health, heatwaves are classified by three grades according to the magnitude of the HI, as listed in Table 2.

**Table 2:** Classification standard of heatwaves.

Grade	Heatwave index	Description
Light heatwave	2.8 ≤ HI < 6.5	A certain impact on public health and social-economic activities
Moderate heatwave	6.5 ≤ HI < 10.5	A relatively serious impact on public health and social-economic activities
Strong heatwave	HI ≥ 10.5	A serious and harmful impact on public health and social-economic activities

### Heatwave metrics

To gain a more holistic perspective of the changes in heatwaves according to previous studies^59,60^, we select five metrics for their quantification (Table 3).

**Table 3:** Heatwave metrics.

Metric shorthand	Metric name	Metric definition
HWF	Heatwave frequency	Total number of the heatwaves in a season
HWD	Heatwave duration	Total number of contiguous days from start to end of the heatwave
HWS	Heatwave season length	Number of days from the first to the last heatwave in any given year
HWFT	First heatwave timing	Timing of the first heatwave day in a season
HWLT	Last heatwave timing	Timing of the last heatwave day in a season

### Population exposure to heatwaves (PEH)

PEH is defined as the exposure of population in heatwave-prone areas. That is, PEH can be computed by multiplying the heatwave days and the population for each grid^61^, where the units of exposure are person-days. The spatial resolution of PEH was set at 0.25° × 0.25°.

Changes in the PEH are determined not only by the magnitude and spatial distribution of population but also by climate change^62^. We adopt the method from Jones et al.^19^ to measure the impacts of climate and population on the PEH. The effects of changes in the PEH are divided into three parts.

1. The climate effect, which allows heatwave days to change over time but leaving the population fixed at the base period level.
2. The population effect, which allows the population to change but leaving heatwave days fixed at the base period level.
3. The interactive effect, which is defined as the total exposure change minus the summation of the changes in climate and population.

To facilitate comparison, we select the interval from 2001 to 2005 as the base period (T0). The comparison periods are 2006–2010 (T1), 2011–2015 (T2), and 2016–2020 (T3), respectively.

The decomposition for PEH changes is calculated according to Eq. (7)

7 $$\Delta {\text{E}}_{{{\text{pop}}}} = {\text{P}}_{{\text{j}}} \times {\text{C}}_{{\text{j}}} - {\text{P}}_{{\text{i}}} \times {\text{C}}_{{\text{i}}} = {\text{P}}_{{\text{i}}} \times \Delta {\text{C}} + {\text{C}}_{{\text{i}}} \times \Delta {\text{P}} + \Delta {\text{P}} \times \Delta {\text{C}}$$

where ${\Delta E}_{pop}$ represents the total change in PEH. ${P}_{i}$ and ${C}_{i}$ represent the population and the number of heatwave days in period $i$, respectively. ${P}_{j}$ and ${C}_{j}$ represent the population and the number of heatwave days in period ${\text{j}}$, respectively. ∆${\text{P}}$ and ∆${\text{C}}$ represent the changes in population and the number of heatwave days from period $i$ to period ${\text{j}}$, respectively. ${\text{P}}_{\text{i}} \cdot \Delta{\text{C}}$ represents the climate effect, ${\text{C}}_{\text{i}} \cdot \Delta{\text{P}}$ represents the population effect, and $\Delta{\text{P}} \cdot \Delta{\text{C}}$ represents the interactive effect (Supplementary information).

Therefore, the contribution rate of each factor is calculated according to:

8 $${\text{CR}}_{{{\text{cli}}}} = \frac{{\left| {{\text{P}}_{{\text{i}}} \times \Delta {\text{C}}} \right|}}{{\left| {{\text{P}}_{{\text{i}}} \times \Delta {\text{C}}} \right| + \left| {{\text{C}}_{{\text{i}}} \times \Delta {\text{P}}} \right| + \left| {\Delta {\text{P}} \times \Delta {\text{C}}} \right|}} \times 100\%$$

9 $${\text{CR}}_{{{\text{pop}}}} = \frac{{\left| {{\text{C}}_{{\text{i}}} \times \Delta {\text{P}}} \right|}}{{\left| {{\text{P}}_{{\text{i}}} \times \Delta {\text{C}}} \right| + \left| {{\text{C}}_{{\text{i}}} \times \Delta {\text{P}}} \right| + \left| {\Delta {\text{P}} \times \Delta {\text{C}}} \right|}} \times 100\%$$

10 $${\text{CR}}_{{{\text{cli}} + {\text{pop}}}} = \frac{{\left| {\Delta {\text{P}} \times \Delta {\text{C}}} \right|}}{{\left| {{\text{P}}_{{\text{i}}} \times \Delta {\text{C}}} \right| + \left| {{\text{C}}_{{\text{i}}} \times \Delta {\text{P}}} \right| + \left| {\Delta {\text{P}} \times \Delta {\text{C}}} \right|}} \times 100\%$$

where ${\text{CR}}_{\text{cli}}$ refers to the contribution rate of the climate effect, ${\text{CR}}_{\text{pop}}$ represents the contribution rate of the population effect, and ${\text{CR}}_{\text{cli+pop}}$ is the contribution rate of the interactive effect.

### Supplementary Information

Supplementary Information.

## Data Availability

The data that support the findings of this study are available from the corresponding author upon reasonable request.
