# Author Correction: Historic evolution of population exposure to heatwaves in Xinjiang Uygur Autonomous Region, China

**DOI:** 10.1038/s41598-024-58100-z

**Published:** 2024-03-27

**Authors:** Diwen Dong, Hui Tao, Zengxin Zhang

**Affiliations:** 1 College of Ecology and Environment, Xinjiang University, Urumqi, 830046 China; 2 State Key Laboratory of Desert and Oasis Ecology, Xinjiang Institute of Ecology and Geography, Chinese Academy of Sciences, Urumqi, 830011 China; 3 University of Chinese Academy of Sciences, Beijing, 100049 China; 4 College of Statistics & Data Science, Xinjiang University of Finance & Economics, Urumqi, 830012 China; 5 Joint Innovation Center for Modern Forestry Studies, College of Forestry, Nanjing Forestry University, Nanjing, 210037 Jiangsu China

Correction to: *Scientific Reports* 10.1038/s41598-023-34123-w, published online 06 May 2023

In the original version of this Article, the Affiliations were not listed in the correct order. The correct affiliations are listed below:

Affiliation 1:

College of Ecology and Environment, Xinjiang University, Urumqi, 830046, China

Affiliation 2:

State Key Laboratory of Desert and Oasis Ecology, Xinjiang Institute of Ecology and Geography, Chinese Academy of Sciences, Urumqi, 830011, China

Affiliation 3:

University of Chinese Academy of Sciences, Beijing, 100049, China

Affiliation 4:

College of Statistics & Data Science, Xinjiang University of Finance & Economics, Urumqi, 830012, China

Affiliation 5:

Joint Innovation Center for Modern Forestry Studies, College of Forestry, Nanjing Forestry University, Nanjing, 210037, Jiangsu, China

The original Article has been corrected.

